# Active ageing behaviors among urban older adults in disaster-prone communities using confirmatory factor analysis of health behavior constructs

**DOI:** 10.1038/s41598-026-46240-3

**Published:** 2026-04-06

**Authors:** Weerayut Muenboonme, Pachanat Nunthaitaweekul, Bhichit Rattakul, Welawat Tienpratarn

**Affiliations:** 1 Department of Disaster and Emergency Medical Operation, Faculty of Science and Health Technology, Navamindradhiraj University, Bangkok, 10300 Thailand; 2 Faculty of Nursing, Chulalongkorn University, Bangkok, 10330 Thailand; 3 Asian Disaster Preparedness Center (ADPC), Bangkok, 10400 Thailand; 4 Thai Network for Disaster Resilience (TNDR), Bangkok, 10300 Thailand; 5 Department of Emergency Medicine, Faculty of Medicine, Ramathibodi Hospital, Mahidol University, 270 Rama VI Road, Thung Phaya Thai, Ratchathewi, Bangkok, 10400 Thailand

**Keywords:** Active ageing, Health behavior, Older adults, Urban ageing, Disaster-prone areas, Confirmatory factor analysis, Behavioral resilience, Thailand

## Abstract

Older adults in disaster-prone urban areas face elevated risks from environmental hazards, chronic disease, and social vulnerabilities. Although active ageing is widely promoted, limited evidence has validated its behavioral domains in hazard-prone urban contexts. This study aimed to examine the factorial structure of active ageing behaviors and identify sociodemographic influences among older adults in Bangkok, Thailand. A cross-sectional survey was conducted using a multistage sampling design, involving purposive selection of disaster-prone districts followed by random sampling at subsequent stages, among 500 older adults in Bangkok. Data were collected through structured, interviewer-administered questionnaires based on the WHO Active Ageing Framework, covering six domains. Confirmatory Factor Analysis (CFA) and Generalized Structural Equation Modeling (GSEM) were performed using maximum likelihood estimation with robust standard errors. CFA supported a four-factor model comprising dietary behavior, stress management, self-care, and substance avoidance with significant loadings (β = 0.711–1.095) and excellent fit (CFI = 0.991, TLI = 0.972, RMSEA = 0.057, SRMR = 0.020). Substance avoidance and stress management were most salient. The six-factor model including exercise and oral care showed poor fit. GSEM indicated that male sex was negatively associated with active ageing (β =  − 0.23, *p* < 0.001), while higher education, cohabitation, and chronic disease were positive predictors. The validated four-domain model highlights active ageing as both behavioral and socially embedded. Strengthening behavioral resilience and addressing social and environmental barriers are essential for interventions and policies in disaster-prone urban ageing populations.

**Supplementary Information:**

The online version contains supplementary material available at 10.1038/s41598-026-46240-3.

## Introduction

Global population ageing has intensified demand for strategies that sustain health and independence in later life, especially in urban environments where older adults encounter unique vulnerabilities. The World Health Organization (WHO) defines active ageing as the process of optimizing health, participation, and security to enhance quality of life as people age^1–3^. This concept encompasses behaviors such as healthy diet, physical activity, stress management, avoidance of tobacco and alcohol, oral hygiene, and self-care^4–6^.

Older adults living in large metropolitan areas are increasingly exposed to environmental risks such as floods, air pollution, and heatwaves, which interact with chronic disease and mobility limitations to heighten vulnerability^7–9^. Bangkok, Thailand, exemplifies this challenge as a rapidly urbanizing city that has experienced significant flooding events in 1994, 1995, 2006, and most notably in 2011^10^. Although these events are episodic rather than strictly “recurring,” their scale and impact underscore the city’s susceptibility to hydro-meteorological hazards. In addition, Bangkok consistently records high levels of air pollution, with seasonal peaks of fine particulate matter (PM2.5) exceeding WHO guidelines and posing major health risks to urban residents^11,12^. Older adults in such contexts are disproportionately affected during emergencies due to frailty, pre-existing health conditions, and reduced adaptive capacity^13^. These hazard characteristics informed the selection of study districts, ensuring that the empirical validation of active ageing behaviors was grounded in objectively defined, high-risk urban environments. In disaster-prone urban contexts, active ageing behaviors take on a distinct functional role compared with those in non-hazard settings. Rather than primarily supporting health restoration or optimization under stable conditions, such behaviors are increasingly oriented toward maintaining continuity of daily functioning, mitigating risk exposure, and sustaining self-regulation under recurrent environmental stressors. In contrast, in non-disaster or low-risk settings, active ageing behaviors are typically emphasized as part of long-term health promotion and recovery processes, supported by relatively stable infrastructure and service accessibility. This distinction underscores the need to conceptualize active ageing in hazard-prone cities as a form of behavioral resilience, rather than solely as a health-enhancing lifestyle.

Previous studies on ageing populations have primarily examined individual health behaviors in isolation, without systematically testing their interrelationships as part of a broader construct^14–16^. Few studies have applied rigorous psychometric methods, such as Confirmatory Factor Analysis (CFA) or Structural Equation Modeling (SEM), to validate the factorial structure of active ageing behaviors in hazard-prone urban contexts. This gap in evidence limits the ability to design targeted interventions that strengthen “behavioral resilience” the capacity to maintain health-promoting practices under adverse conditions^17–19^. Importantly, much of the existing literature on active ageing has been generated in non-disaster or relatively stable settings, where environmental disruptions are minimal and health behaviors are primarily framed as mechanisms for recovery, optimization, or quality-of-life enhancement. Consequently, the applicability of these behavioral constructs to disaster-prone urban environments where older adults must sustain health practices amid recurrent hazards, service disruptions, and environmental constraints remains insufficiently examined.

This study addresses these gaps by validating the factorial structure of six domains of active ageing behaviors dietary behavior, exercise, stress management, substance avoidance, oral care, and self-care among older adults in selected high-risk districts of Bangkok. By clarifying the structural domains and identifying key sociodemographic determinants, this research aims to strengthen the evidence base for health promotion and disaster preparedness policies tailored to vulnerable urban ageing populations.

## Methods

### Study design

This study employed a cross-sectional, community-based design to examine the structure of active ageing behaviors among older adults in disaster-prone urban areas. Confirmatory Factor Analysis (CFA) was used to validate the hypothesized six-factor model of behavioral domains within the active ageing construct.

### Setting and participants

The study was conducted in five administrative districts of Bangkok, Thailand. These districts were classified as disaster-prone based on official hazard risk profiles issued by the Bangkok Metropolitan Administration (BMA) and national agencies. Specifically, district-level flood risk maps, historical flood records (including the 2011 mega-flood), PM2.5 air pollution surveillance data, and urban heat risk indicators were reviewed. Districts with recurrent exposure to at least two major urban hazards flooding, air pollution, or heat stress were purposively selected to represent high-risk urban ageing contexts. Eligible participants were community-dwelling adults aged 60 years or older, residing in the selected districts for at least one year. Inclusion criteria included the ability to communicate in Thai and a score of ≥ 8 on the Thai version of the Abbreviated Mental Test. Individuals with severe illness or cognitive impairment were excluded.

A multistage sampling design was employed. In the first stage, districts were purposively selected based on predefined disaster-prone criteria (e.g., recurrent flooding, high-density urban settlement, and documented environmental risk exposure). In subsequent stages, subdistricts and eligible participants were selected using random sampling procedures to ensure representativeness within the targeted areas. This approach enabled targeted inclusion of environmentally vulnerable urban contexts while maintaining random selection procedures at the participant level.

### Sampling procedures

The multistage sampling design consisted of three sequential stages. First, disaster-prone districts were purposively selected based on official hazard risk profiles, including flood exposure, PM2.5 air pollution surveillance data, and urban heat risk indicators. Second, communities (subdistricts) within each selected district were randomly sampled. Third, eligible older adults were randomly selected from community health records maintained by local health authorities.

A multistage sampling approach was applied. Purposive selection was employed to ensure inclusion of districts with documented, multi-hazard exposure, as defined by official flood, air pollution, and heat-risk assessments. This approach was chosen to enhance contextual relevance rather than population representativeness. Second, communities within each district were randomly chosen. Finally, eligible individuals were randomly selected from local health records. A sample size of 500 was determined based on CFA recommendations (≥ 10 participants per parameter) and to ensure model stability.

In the final CFA model, a total of 9 free parameters were estimated, including three factor loadings, four measurement error variances, one latent variance, and one latent mean. In the subsequent GSEM, an additional four structural regression paths from sociodemographic predictors to the latent active ageing construct were estimated, resulting in approximately 13 free parameters in the full model. Based on the recommended ratio of at least 10 participants per estimated parameter, a minimum sample size of 130 would have been sufficient. Therefore, the achieved sample size of 500 participants was more than adequate to ensure model stability and reliable parameter estimation.

Thus, the achieved sample size substantially exceeded conventional CFA and SEM recommendations, supporting stable parameter estimation and overall model robustness.

### Measures

A structured questionnaire was developed based on the WHO Active Ageing Framework and relevant literature. It included six domains: Food (6 items), Exercise (2 items), No stress (2 items), No smoking and alcohol (4 items), Dental care (3 items) and Self-care (5 items). Items were rated on a 5-point Likert scale (1 = never, 5 = always). Content validity was reviewed by public health and ageing experts. Internal consistency (Cronbach’s alpha) ranged from 0.76 to 0.88. Given that CFA was conducted using domain-level composite scores rather than item-level indicators, composite reliability (CR) and average variance extracted (AVE) were not separately estimated. Internal consistency and standardized factor loadings were used to evaluate measurement adequacy at the domain level. A pilot test was conducted with 30 older adults, and minor revisions were made for clarity and cultural relevance. This questionnaire was previously developed and validated in prior published studies^19,20^. The English version focused on active ageing is provided as Supplementary File 1.

### Data collection

Data collection was conducted between January and June 2023. Trained interviewers administered face-to-face surveys in participants’ homes or designated community locations. Daily supervision ensured completeness, consistency, and data quality throughout the fieldwork process.

### Statistical analysis

Prior to CFA and GSEM analyses, data diagnostics were conducted to assess key modeling assumptions. Univariate distributions were examined for outliers and extreme values, with no observations exceeding ± 3 standard deviations. Correlation matrices were inspected to assess linearity and multicollinearity, with all inter-factor correlations below 0.70. Internal consistency across domains was acceptable (Cronbach’s α = 0.76–0.88), supporting scale reliability.

Missing data were minimal (< 5%) and were handled using full information maximum likelihood (FIML), which provides unbiased parameter estimates under the missing-at-random assumption.

Descriptive statistics were used to summarize participant characteristics and behavior scores. Independent t-tests and one-way ANOVA were conducted to examine group differences. CFA was performed using maximum likelihood estimation with robust standard errors. These preliminary analyses were conducted to explore unadjusted group differences in active ageing behaviors and to provide descriptive context for subsequent multivariable modeling. The results were not intended to establish independent effects but to inform and complement the GSEM analysis.

Although the questionnaire items were ordinal, CFA was conducted using robust maximum likelihood estimation based on domain-level composite scores. These composite indicators approximate continuous distributions and demonstrated acceptable internal consistency and stable variances. Robust standard errors were applied to account for potential deviations from multivariate normality. This approach has been commonly adopted in CFA studies examining higher-order behavioral constructs.

Given the multidimensional structure of the questionnaire and the varying number of items across domains, CFA was conducted using domain-level composite scores rather than individual items. This approach was adopted to reduce model complexity, enhance parameter stability, and ensure comparability across behavioral domains. Composite scores were calculated as the mean of items within each domain, all of which demonstrated acceptable internal consistency (Cronbach’s α = 0.76–0.88). This strategy is consistent with previous CFA studies examining higher-order behavioral or lifestyle constructs, where domains are treated as theoretically meaningful indicators of a latent construct. Accordingly, the resulting measurement model should be interpreted as reflecting higher-order behavioral domains derived from composite indicators, rather than a fully item-level latent structure. While this approach enhances model parsimony and stability, it limits the ability to examine item-level factor loadings and formal measurement invariance across subgroups.

Model fit was evaluated using χ^2^, Comparative Fit Index (CFI), Tucker-Lewis Index (TLI), Root Mean Square Error of Approximation (RMSEA), and Standardized Root Mean Square Residual (SRMR). Acceptable thresholds were defined as: CFI and TLI ≥ 0.90, RMSEA ≤ 0.08, and SRMR ≤ 0.08.

Multiple fit indices were selected to capture complementary aspects of model fit, including absolute fit (χ^2^, SRMR), incremental fit (CFI, TLI), and parsimony-adjusted fit (RMSEA), in accordance with established SEM guidelines.

For model identification, one factor loading was fixed to unity, and variance estimates were examined to ensure admissible solutions without negative residuals.

For model identification, one factor loading was fixed to unity for the latent construct, while remaining loadings and variance parameters were freely estimated. All solutions were admissible, with no negative variance estimates or convergence problems observed.

Model refinement was conducted following established CFA guidelines. When the hypothesized six-factor model demonstrated inadequate fit, factor loadings, residuals, and modification indices were examined. Domains with consistently low standardized loadings (< 0.40), limited internal coherence, or conceptual overlap were considered for removal. Model re-specification was guided by both statistical criteria and theoretical interpretability, with preference given to parsimonious structures that retained conceptual alignment with the WHO Active Ageing Framework.

Following the CFA, Generalized Structural Equation Modeling (GSEM) was employed to examine the associations between the latent active ageing construct and multiple sociodemographic predictors within a single analytical framework. GSEM was selected to allow simultaneous estimation of measurement and structural components while accommodating mixed distributions of endogenous variables and providing robust standard errors. Although most observed indicators were continuous, GSEM offered greater flexibility in modeling complex relationships and ensured consistency with the CFA-based latent structure.

CFA and GSEM analyses were conducted using robust maximum likelihood estimation to evaluate the measurement and structural components of the proposed model. In the final CFA and GSEM models, the number of estimated free parameters was limited relative to the sample size.

The ratio of sample size to estimated parameters substantially exceeded conventional CFA and SEM recommendations, further supporting the robustness of the analytical results.

Analyses were conducted using STATA version 17.0 (StataCorp LLC, TX, USA).

## Results

### Statistical analysis of sample

To evaluate the data collected from respondents (N = 500), all statistical analyses were conducted using STATA 17.0 (StataCorp LLC, College Station, TX, USA). Descriptive statistics were used to summarize the characteristics of the study population.

Prior to conducting the Confirmatory Factor Analysis (CFA), independent sample t-tests and one-way analysis of variance (ANOVA) were performed to examine differences in active ageing behaviors across demographic and socio-economic groups. The t-test was used to compare mean differences between two independent groups, such as sex (male vs. female) and age groups (< 70 years vs. ≥ 70 years), while one-way ANOVA was employed to assess variations across multiple categorical groups, including education level. These findings provide an initial overview of group-level variations and serve as descriptive evidence that is further examined within the integrated GSEM framework.

Following these preliminary analyses, CFA utilizing Structural Equation Modeling (SEM) was applied to assess the Active Ageing in Urban Communities During Disasters model. The model’s goodness-of-fit was determined using multiple indices, including chi-square (χ^2^), comparative fit index (CFI), Tucker-Lewis index (TLI), and root mean square error of approximation (RMSEA). A non-significant χ^2^ suggests an adequate model fit. Standard criteria for acceptable model fit include CFI and TLI values above 0.90 and RMSEA below 0.08.

These analyses provided a comprehensive evaluation of the relationships between demographic characteristics and active ageing behaviors, as well as the structural validity of the proposed model. Detailed descriptive statistics, including means and standard deviations across key sociodemographic groups, are reported in Table 1 to support transparency and reproducibility of the reported findings.

**Table 1:** General characteristics of elderly populations in the community and mean scores of 6 categories of active ageing behaviors.

Variables	N (%) (n = 500)	Food	Exercise	No stress	No smoking and alcohol	Dental care	Self-care
Sex							
Male	189 (37.80)	3.44 ± 0.85 (*)	3.31 ± 1.27	3.31 ± 1.15	3.87 ± 1.27 (*)	3.92 ± 0.84	4.07 ± 0.72
Female	311 (62.20)	3.61 ± 0.81 (*)	3.23 ± 1.27	3.49 ± 1.10	4.52 ± 0.87 (*)	3.95 ± 0.76	4.13 ± 0.68
Age (years) (Mean ± SD)	68.61 ± 7.31						
Age < 70 years	315 (63.00)	3.50 ± 0.83	3.29 ± 1.07	3.49 ± 1.10	4.18 ± 1.13 (*)	3.92 ± 0.77	4.09 ± 0.69
Age ≥ 70 years	185 (37.00)	3.63 ± 0.83	3.21 ± 1.29	3.29 ± 1.15	4.44 ± 0.99 (*)	3.98 ± 0.82	4.14 ± 0.69
Marriage status							
Married	234 (46.80)	3.52 ± 0.81	3.42 ± 1.08 (*)	3.47 ± 1.12	4.16 ± 1.10 (*)	3.89 ± 0.76	4.12 ± 0.69
Single/others	266 (53.20)	3.57 ± 0.85	3.11 ± 1.21 (*)	3.37 ± 1.12	4.38 ± 1.07 (*)	3.98 ± 0.81	4.09 ± 0.69
Type of family							
Single family	388 (77.60)	3.48 ± 0.86 (*)	3.29 ± 1.13	3.39 ± 1.09	4.24 ± 1.13	3.97 ± 0.78	4.08 ± 0.68
Extended family	112 (22.40)	3.77 ± 0.68 (*)	3.14 ± 1.26	3.53 ± 1.21	4.40 ± 0.90	3.86 ± 0.81	4.19 ± 0.73
Living arrangement							
Living alone	83 (16.60)	3.40 ± 0.93	3.24 ± 1.31	3.04 ± 1.18 (*)	4.01 ± 1.40 (*)	4.13 ± 0.76 (*)	3.96 ± 0.72 (*)
Reside with others	417 (83.40)	3.57 ± 0.81	3.26 ± 1.13	3.50 ± 1.09 (*)	4.33 ± 1.01 (*)	3.90 ± 0.79 (*)	4.14 ± 0.68 (*)
Level of education*							
Low	388 (77.60)	3.52 ± 0.85	3.28 ± 1.14	3.37 ± 1.12 (*)	4.29 ± 1.09	3.97 ± 0.79	4.08 ± 0.67
Middle	83 (16.60)	3.58 ± 0.76	3.10 ± 1.26	3.39 ± 1.11 (*)	4.12 ± 1.11	3.80 ± 0.76	4.20 ± 0.71
High	29 (5.80)	3.80 ± 0.74	3.43 ± 1.05	4.16 ± 0.93 (*)	4.52 ± 0.90	4.02 ± 0.90	4.24 ± 0.87
Chronic disease							
Yes	311 (62.20)	3.65 ± 0.77 (*)	3.11 ± 1.18 (*)	3.31 ± 1.20	4.45 ± 0.87 (*)	3.90 ± 0.79	4.19 ± 0.67 (*)
No	189 (37.80)	3.37 ± 0.90 (*)	3.50 ± 1.08 (*)	3.49 ± 1.06	3.99 ± 1.32 (*)	4.01 ± 0.79	3.96 ± 0.70 (*)
Community member**							
Non-active	428 (85.60)	3.55 ± 0.82	3.23 ± 1.15	3.42 ± 1.11	4.27 ± 1.10	3.92 ± 0.80	4.06 ± 0.69 (*)
Active	72 (14.40)	3.54 ± 0.92	3.46 ± 1.17	3.44 ± 1.19	4.31 ± 0.99	4.06 ± 0.74	4.41 ± 0.63 (*)
Occupation							
Employed	148 (29.60)	3.64 ± 0.78	3.32 ± 0.96	3.72 ± 1.00 (*)	4.38 ± 0.97	3.85 ± 0.83	4.17 ± 0.73
Not employed	352 (70.40)	3.50 ± 0.85	3.24 ± 1.23	3.30 ± 1.15 (*)	4.23 ± 1.13	3.98 ± 0.77	4.08 ± 0.67

* Level of education: Low (no formal education, primary education, lower secondary education), Middle (upper secondary education, vocational certificate, associate degree), High (bachelor’s degree, postgraduate degree).

** Community member: Non-active (general public), Active (volunteers, village health volunteers, community committee members).

(*) *P*-value < 0.05.

### General characteristics of the elderly population and active ageing behaviors

The study examined 500 elderly individuals in the community and assessed their active ageing behaviors across six categories: food behavior, exercise, no stress behavior, no smoking and alcohol behavior, dental care, and self-care behavior. The mean scores for each category were compared across different demographic and socio-economic characteristics (Table 1).

The analysis of sex differences in active ageing behaviors revealed that females exhibited significantly higher scores in food behavior (3.61 ± 0.81, *p* < 0.05) and no smoking & alcohol behavior (4.52 ± 0.87, *p* < 0.05) compared to males. Although females also demonstrated slightly higher self-care behavior scores (4.13 ± 0.68) than males (4.07 ± 0.72), this difference was not statistically significant.

Regarding age groups, elderly individuals aged ≥ 70 years had higher scores in food behavior (3.63 ± 0.83) and no smoking & alcohol behavior (4.44 ± 0.99, *p* < 0.05) compared to those younger than 70 years. However, self-care behavior scores remained comparable between the two age groups.

Marital status appeared to influence certain active ageing behaviors, as married elderly individuals reported significantly higher exercise behavior (3.42 ± 1.08, *p* < 0.05) but lower dental care scores (3.89 ± 0.76, *p* < 0.05) compared to those who were single or in other marital categories.

Family type and living arrangements were also associated with variations in active ageing behaviors. Elderly individuals residing in extended families reported higher food behavior scores (3.77 ± 0.68, *p* < 0.05) but exhibited lower exercise behavior (3.14 ± 1.26) than those in single-family households. Additionally, elderly individuals who lived alone demonstrated significantly lower scores in no stress behavior (3.04 ± 1.18, *p* < 0.05) and dental care (4.01 ± 1.40, *p* < 0.05) compared to those who lived with others.

Education level played a role in shaping active ageing behaviors, as individuals with higher education levels exhibited significantly better self-care behavior (4.24 ± 0.87, *p* < 0.05) and higher no stress behavior scores (4.16 ± 0.93, *p* < 0.05) than those with lower education levels.

Health conditions and community engagement were also found to be influential. Elderly individuals with participants with chronic disease reported significantly lower exercise behavior (3.11 ± 1.18, *p* < 0.05) and no stress behavior (3.31 ± 1.20, *p* < 0.05) scores compared to those without chronic disease. Moreover, those who were actively involved in community activities demonstrated significantly better self-care behavior (4.41 ± 0.63, *p* < 0.05) than their non-active counterparts.

Lastly, employment status was found to impact stress management, with employed elderly individuals reporting significantly higher no stress behavior scores (3.72 ± 1.00, *p* < 0.05) than those who were unemployed. These findings highlight the importance of demographic, social, and economic factors in influencing active ageing behaviors among the elderly population.

### Correlation analysis

This study examined the relationships among six key components of active ageing behaviors: food, exercise, no stress, smoking and alcohol, dental care, and self-care. The significance level for all correlations was set at *p* < 0.05, with statistically significant relationships denoted by an asterisk (*).

A correlation analysis was conducted to assess the potential for multicollinearity among these variables, as excessive correlation can lead to biased estimations in structural equation modeling. The correlation coefficients ranged from − 0.24 to 0.44, indicating mild to moderate associations among the variables.

**Fig. 1:**
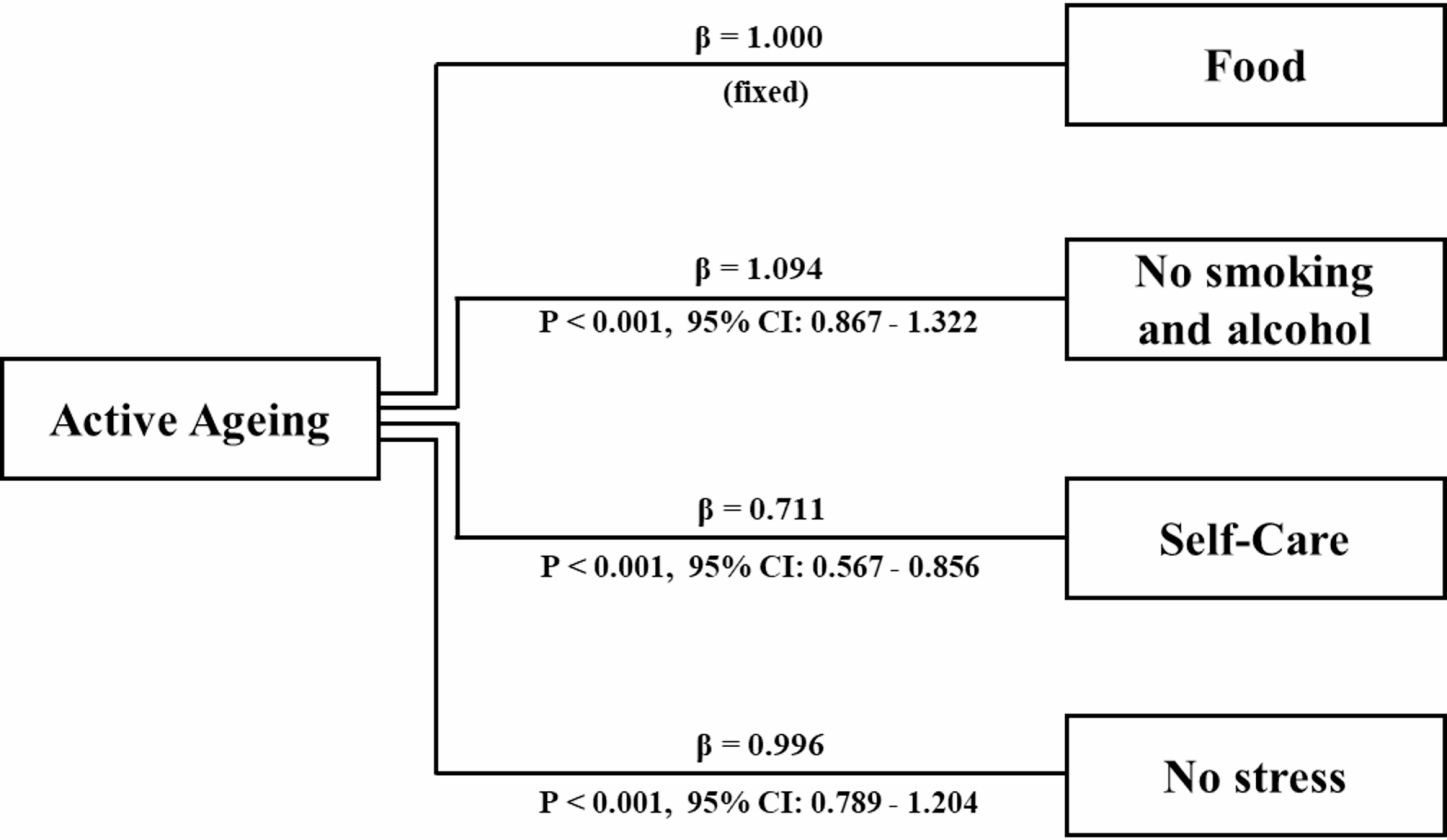
Standardized regression weights for active ageing behaviors model.

As detailed in Fig. 1, self-care demonstrated a moderate positive correlation with food (r = 0.44) and no smoking and alcohol (r = 0.41). Additionally, no stress negatively correlated with no smoking & alcohol (r = 0.30). In contrast, exercise showed a weak negative correlation with no stress (r = − 0.24).

Since none of the correlation coefficients exceeded 0.70, concerns regarding multicollinearity were minimal. Therefore, all independent variables were retained in the structural model. These results suggest that while each factor is interrelated, they contribute distinctively to the overall construct of active ageing. These results further support the suitability of the data for structural equation modeling. The full correlation matrix among all observed behavior domains is provided in Table 2 to facilitate transparency and reproducibility of the measurement structure.

**Table 2:** Correlation matrix between 6 behavior categories of active ageing behaviors mean score (food, exercise, no stress, no smoking and alcohol, dental care, and self-care).

Variables	Food	Exercise	No stress	No smoking and alcohol	Dental care	Self-care
Food	1.00	− 0.18*	0.42*	0.42*	− 0.03	0.44*
Exercise	− 0.18*	1.00	− 0.24*	− 0.16	0.17*	− 0.00
No stress	0.42*	− 0.24*	1.00	0.30*	− 0.11	0.30*
No smoking and alcohol	0.42*	− 0.16	0.30*	1.00	0.07	0.41*
Dental care	− 0.03	0.17*	− 0.11	0.07	1.00	0.17*
Self-care	0.44*	− 0.00	0.30*	0.41*	0.17*	1.00

* *P*-value < 0.05.

### Confirmatory factor analysis (CFA)

The CFA was initially performed to test the hypothesized six-factor structure of active ageing behaviors. However, the six-factor model demonstrated inadequate fit to the data (see Section “Model fit for six-factor structure”). Consequently, a refined model was specified by excluding the exercise and dental care domains, resulting in a four-factor structure that was subsequently evaluated.

The confirmatory factor analysis (CFA) was conducted to assess the structural validity of the active ageing behaviors model, which is conceptualized as a latent construct measured by four observed domain-level composite indicators: food behavior, no smoking and alcohol behavior, self-care behavior, and no stress behavior. It should be noted that each observed indicator represents a domain-level composite score calculated as the mean of multiple items. Therefore, the CFA results reflect relationships between higher-order behavioral domains and the latent construct of active ageing, rather than direct item-factor associations. Maximum likelihood estimation with robust standard errors was employed to ensure the stability of parameter estimates.

As detailed in Table 2, All factor loadings were statistically significant (*p* < 0.05), indicating strong relationships between the latent construct and its indicators. Specifically, no smoking & alcohol behavior exhibited the highest factor loading (β = 1.095), followed by no stress behavior (β = 0.996) and self-care behavior (β = 0.711). Food behavior was fixed at 1.000 for model identification.

One standardized loading slightly exceeded unity (β = 1.095). This phenomenon may occur in models employing composite indicators due to scaling characteristics and strong inter-factor correlations, rather than indicating model misspecification. Examination of residual variances confirmed that no negative variance estimates were observed, and overall model fit indices remained within acceptable thresholds. Therefore, this estimate is interpreted cautiously as reflecting a strong association between the latent construct and the composite indicator rather than a statistical anomaly.

The variance estimates of the observed variables were within an acceptable range, with self-care behavior (0.296, SE = 0.030) and food behavior (0.328, SE = 0.043) exhibiting lower variance than no smoking & alcohol behavior (0.749, SE = 0.080) and no stress behavior (0.895, SE = 0.068). The variance of the latent construct active ageing was statistically significant (0.359, SE = 0.047), confirming that the model adequately captures variability in active ageing behaviors.

Overall, the model demonstrated an acceptable fit to the data, with CFI = 0.991, TLI = 0.972, RMSEA = 0.057, and SRMR = 0.020, suggesting that the proposed measurement structure reasonably represents the observed relationships among active ageing behaviors.

### Generalized structural equation model (GSEM)

In this analysis, active ageing was specified as the latent construct, reflected through multiple observed health behavior domains.

Building upon the validated CFA measurement model, GSEM was applied to simultaneously assess the relationships between active ageing and sociodemographic determinants.

The generalized structural equation model (GSEM) indicated that the latent active ageing construct was significantly associated with all observed health behavior domains (Table 3). The strongest effect was identified for avoidance of smoking and alcohol consumption (β = 1.19, SE = 0.12, z = 9.72, *p* < 0.001), followed by stress management (β = 1.03, SE = 0.11, z = 9.50, *p* < 0.001) and self-care (β = 0.73, SE = 0.07, z = 10.12, *p* < 0.001). These results suggest that behavioral regulation and coping-related domains demonstrate the strongest associations within the active ageing construct.

**Table 3:** Standardized path coefficients of generalized structural equation model (GSEM) for predictors of active ageing and related outcomes.

Outcome	Predictor	β	SE	z	*p*-value
Food	Active ageing	1 (ref.)	–	–	–
No smoking and alcohol		1.19	0.12	9.72	< 0.001
Self-Care		0.73	0.07	10.12	< 0.001
No stress		1.03	0.11	9.50	< 0.001
Active ageing	Male	− 0.23	0.06	-3.66	< 0.001
	Chronic disease	0.27	0.06	4.29	< 0.001
	Level of education	0.14	0.05	2.68	0.007
	Living arrangement	0.19	0.08	2.44	0.014

*β* coefficients, *SE* standard error, *z* z-statistic.

In addition to health behaviors, several sociodemographic predictors were statistically significant. Male participants were less likely to demonstrate active ageing behaviors (β = − 0.23, SE = 0.06, z = − 3.66, *p* < 0.001). Conversely, chronic disease (β = 0.27, SE = 0.06, z = 4.29, *p* < 0.001), higher educational attainment (β = 0.14, SE = 0.05, z = 2.68, *p* = 0.007), and living arrangements with others (β = 0.19, SE = 0.08, z = 2.44, *p* = 0.014) were positively associated with active ageing.

Overall, the GSEM results complement the CFA findings by providing evidence of both behavioral and sociodemographic determinants of active ageing. These findings suggest that active ageing in disaster-prone urban settings is not solely shaped by individual health practices but is also influenced by broader social and demographic conditions.

### Model fit for six-factor structure

The model specifying Active Ageing as a latent construct with six observed indicators, Food Behavior, Self-Care, No Smoking and Alcohol Behavior, Stress Avoidance, Dental Care, and Exercise Behavior, showed poor fit. The chi-square test was significant (χ^2^(9) = 75.867, *p* < 0.001), and fit indices did not meet acceptable thresholds (RMSEA = 0.122, CFI = 0.846, TLI = 0.743). Although SRMR (0.074) and CD (0.733) suggested moderate explanatory power, the overall model fit was inadequate, indicating the need for model refinement or adjustment of observed indicators.

Further inspection revealed that the exercise and dental care domains exhibited weak standardized loadings and limited contribution to overall model fit. Given their low explanatory value and to enhance model parsimony, these two domains were excluded from the final measurement model.

The exclusion of exercise and oral care from the final model was guided by both statistical and theoretical considerations. Statistically, both domains demonstrated weak standardized loadings and limited contribution to overall model fit. Theoretically, these behaviors may be more sensitive to contextual or infrastructural conditions in dense urban environments, which were not directly measured in the present study.

All path coefficients were statistically significant (*p* < 0.05), suggesting that active ageing is a strong predictor of all four observed behaviors.

## Discussion

### Structural validation of active ageing behaviors

This study provides empirical evidence supporting the structural validity of active ageing behaviors among older adults residing in disaster-prone urban communities, within the context and methodological constraints of the present analysis. Confirmatory factor analysis (CFA) confirmed a four-domain model comprising dietary behavior, stress management, self-care, and avoidance of smoking and alcohol. All domains demonstrated significant factor loadings onto the latent construct of active ageing, with overall fit indices reaching recommended thresholds (CFI = 0.991, TLI = 0.972, RMSEA = 0.057, SRMR = 0.020), demonstrating both statistical robustness and theoretical alignment with the WHO Active Ageing Framework. Specifically, all standardized factor loadings were statistically significant (*p* < 0.05), and the overall model demonstrated excellent fit (CFI = 0.991, TLI = 0.972, RMSEA = 0.057, SRMR = 0.020), providing empirical support for the retained four-domain structure.

Within this validated structure, avoidance of smoking and alcohol exhibited the highest standardized loading (β = 1.094), followed by stress management (β = 0.996), self-care (β = 0.711), and dietary behavior (fixed at β = 1.000). The predominance of substance avoidance (β = 1.094) and stress management (β = 0.996) underscores the central position of behavioral regulation and coping capacity within the observed structure of active ageing, as reflected by their strong association with the latent construct^3,21,22^. Although the standardized loading for substance avoidance slightly exceeded 1.0, this estimate should be interpreted cautiously, as it may reflect scaling effects associated with composite domain scores rather than over-identification of the latent construct. This pattern is consistent with previous empirical studies that conceptualize active ageing as a behaviorally regulated construct, in which risk avoidance and emotional regulation represent core dimensions of health maintenance in later life^4,21,22^. Similar factor structures emphasizing substance avoidance and stress-related behaviors have been reported in non-disaster urban settings; however, the relative prominence of these domains in the present study appears more pronounced, potentially reflecting heightened risk awareness and adaptive responses among older adults residing in hazard-prone environments. This comparison suggests that while the foundational components of active ageing remain stable across contexts, their relative salience may vary according to environmental vulnerability. In particular, the emphasis on risk-avoidant behaviors and emotional regulation reflects the heightened vulnerability of urban populations to environmental stressors and disaster-related risks.

The subsequent GSEM analysis extended the CFA findings by identifying several sociodemographic factors that were significantly associated with variations in active ageing.

Male sex was negatively associated with active ageing, while higher educational attainment, cohabitation, and the presence of chronic disease were positively associated within the cross-sectional analytical framework. These results highlight the multidimensional nature of active ageing, demonstrating that it is influenced not only by individual health practices but also by broader demographic and social conditions. Such evidence reinforces the importance of integrating behavioral and contextual determinants when designing interventions to promote active ageing in complex urban environments.

Furthermore, the exclusion of exercise and oral care from the validated structure due to low factor loadings warrants careful theoretical consideration. Two complementary explanations may account for their weaker associations. First, these domains may be contextually influenced by structural and environmental constraints in disaster-prone urban settings, where access to safe physical spaces and preventive dental services may be uneven or disrupted, particularly in high-density metropolitan environments^23,24^. In such contexts, behavioral regulation related to substance avoidance and stress management may assume greater salience in shaping active ageing. Alternatively, the weak loadings may reflect measurement-related factors, including restricted variability, domain operationalization, or limited contextual sensitivity of the composite indicators used. Therefore, the exclusion of these domains should not be interpreted as evidence of their irrelevance to active ageing, but rather as an indication that further refinement of context-sensitive measurement approaches may be necessary in future instrument development.

In sum, the resulting four-domain model offers a contextually relevant and parsimonious framework for understanding active ageing behaviors in disaster-prone urban settings, rather than a definitive or universally generalizable structure. By establishing the structural domains and identifying key sociodemographic influences, the present study contributes to strengthening the evidence base for embedding behavioral resilience within active ageing frameworks in disaster-prone urban settings^25^.

### Sociodemographic influences and health disparities

The GSEM analysis identified sociodemographic characteristics that were significantly associated with active ageing behaviors, reflecting both protective and constraining factors. Male sex was negatively associated with active ageing, consistent with previous evidence that older men are less likely than women to adopt preventive health practices and risk-avoidant behaviors^26,27^. This finding reinforces the persistence of gendered disparities in health engagement and highlights the need for tailored, gender-sensitive interventions.

In contrast, the presence of chronic disease was positively associated with active ageing. Although counterintuitive, this association may indicate compensatory behavioral adaptations, whereby older adults with chronic disease adopt healthier practices in response to increased medical advice and heightened risk perception^28^. This result underscores the importance of integrating behavioral reinforcement into chronic disease management strategies to optimize resilience.

Educational attainment was positively associated with active ageing, aligning with prior research that links education to enhanced health literacy, improved self-regulation, and greater capacity to engage with healthcare services^29,30^. Similarly, living arrangements emerged as a significant determinant: cohabitation was positively associated with active ageing, suggesting that shared routines, mutual support, and access to health-related information enhance behavioral sustainability. These findings are consistent with literature showing that social isolation undermines active ageing behaviors in ageing urban populations^31–33^. From a theoretical perspective, these findings align with social determinants of health and health literacy frameworks, which posit that educational attainment and social embeddedness enhance individuals’ capacity to interpret health information, regulate behavior, and sustain preventive practices over time^29–31^. In urban disaster-prone contexts, such resources may be particularly salient, as social support and knowledge facilitate adaptive coping and continuity of health behaviors under conditions of uncertainty.

Taken together, these results underscore that active ageing is shaped not only by individual health behaviors but also by broader demographic and social determinants. Interventions aiming to promote active ageing in urban, disaster-prone contexts should therefore address structural supports that mitigate gender gaps, capitalize on the adaptive responses of older adults with chronic disease, and strengthen the role of education and social networks in sustaining health behaviors^34,35^.

### Environmental vulnerability and behavioral constraints

It is important to emphasize that environmental exposures, infrastructural conditions, and healthcare accessibility were not directly measured in this study. Therefore, the following interpretations are intended to provide contextual hypotheses grounded in vulnerability and resilience frameworks, rather than causal explanations derived from empirical measurement. The cross-sectional nature of the data further limits any inference regarding directionality or mechanism.

The following interpretations are intended to provide contextual hypotheses that situate the study findings within the broader urban disaster literature, rather than direct inferences derived from measured variables. These observations can be conceptually situated within vulnerability and resilience frameworks, which emphasize the interaction between individual capacities and structural constraints in shaping the behavioral domains of active ageing^36,37^. In this view, active ageing behaviors are not solely determined by personal choice but are conditioned by environmental exposures, infrastructural adequacy, and the availability of supportive services. The present findings therefore resonate with prior studies highlighting the role of urban environmental stressors in shaping adaptive capacity among older adults, while stopping short of implying causal pathways.

The findings indicate that environmental vulnerability may be associated with contextual constraints on the maintenance of active ageing behaviors in disaster-prone urban settings. The relatively weaker structural loading of self-care suggests that consistent engagement in self-care practices may be more challenging in such environments. However, these observations should be interpreted as associations rather than causal effects, as environmental exposures and service accessibility were not directly measured in this cross-sectional study^36,37^.

Infrastructural characteristics in dense urban settings, such as limited access to safe public spaces, inadequate walkways, or insufficient green areas, may be associated with reduced opportunities for physical activity and preventive routines. Although such factors were not directly measured in the present study, they have been reported in prior literature as contextual influences that could plausibly shape behavioral engagement among older adults. These conditions align with the observed lower engagement among older adults living alone, whose lack of immediate social support amplifies the challenges imposed by urban hazards^38^.

Disaster-related disruptions may be associated with challenges in sustaining health behaviors over time; however, these relationships cannot be interpreted as causal pathways given the cross-sectional nature of the data. Service disruptions during flood events or episodes of severe air pollution may be associated with temporary limitations in healthcare access. In such contexts, older adults might shift their focus toward immediate safety concerns, potentially resulting in decreased prioritization of preventive health behaviors. However, these interpretations remain hypothetical, as service accessibility was not directly assessed in this study. Such trade-offs reflect structural inequities in ageing outcomes in urban settings^39,40^.

Chronic exposure to environmental hazards has been associated in prior research with elevated psychological stress, which may influence coping capacity and emotional regulation. While the present study identified stress management as a central behavioral domain, it did not directly measure environmental exposure; therefore, any linkage between hazard exposure and stress-related behaviors should be interpreted cautiously and as contextually informed rather than empirically demonstrated within this dataset. Both domains were shown to be central components of active ageing in this study. Repeated uncertainty and hazard exposure likely contribute to the variability in stress management behaviors across sociodemographic groups^41^.

Collectively, these findings suggest that environmental conditions may constitute important contextual influences on active ageing in disaster-prone urban settings. Nevertheless, given the cross-sectional design and absence of directly measured environmental variables, these relationships should be interpreted as theoretically informed associations rather than confirmed determinants. Structural interventions aimed at improving the urban built environment and mitigating climate-related risks are therefore essential to enable equitable engagement in active ageing behaviors among older adults in disaster-prone urban areas.

While the purposive selection of disaster-prone districts enhances contextual specificity, the findings may be most applicable to high-density metropolitan settings characterized by recurrent environmental stressors, infrastructure strain, and uneven access to preventive health services. Similar urban contexts in Southeast Asia and other rapidly urbanizing regions may exhibit comparable structural conditions. However, extrapolation to rural populations or low-risk urban environments should be undertaken cautiously. Future research should test the stability of this measurement structure across diverse urban typologies, including lower-density municipalities, peri-urban areas, and cities with differing hazard profiles, using multi-site or nationally representative sampling frameworks.

### Integrating behavioral resilience into active ageing frameworks

The validation of active ageing behaviors in this study highlights the need to embed behavioral resilience within ageing frameworks, particularly in disaster-prone urban settings. The structural model confirmed that substance avoidance, stress management, and self-care are critical behavioral domains, while sociodemographic conditions such as sex, education, chronic disease, and living arrangements significantly influence engagement. These results underscore that active ageing is both a behavioral construct and a socially conditioned process, requiring integration across multiple levels of health promotion.

Building resilience into active ageing frameworks involves strengthening individual capacities for behavioral regulation while addressing contextual vulnerabilities. Evidence suggests that health literacy interventions, peer-led support systems, and community-based disaster preparedness may support coping capacity and the maintenance of health practices among older adults facing recurrent hazards^42,43^. At the same time, policies must prioritize structural supports, including safe urban infrastructure, equitable healthcare access, and climate adaptation strategies, to create enabling environments for resilience^44^.

This integration is especially critical in urban communities where environmental risks and social inequalities intersect. Without embedding resilience into the design of ageing policies, older adults may remain disproportionately vulnerable to disruptions in health behaviors during crises. By aligning behavioral promotion with structural interventions, the findings of this study contribute to strengthening the evidence base for active ageing policies that are inclusive, context-sensitive, and sustainable in disaster-prone environments^45,46^.

By situating active ageing within a resilience-oriented framework, this study extends prior conceptualizations that primarily emphasize individual behaviors by explicitly incorporating social and environmental contingencies. In doing so, the findings complement existing WHO-based models by demonstrating how behavioral regulation operates within constrained urban settings, thereby offering a context-sensitive contribution rather than a universal redefinition of active ageing. This integrative perspective strengthens the theoretical relevance of the model for disaster-prone urban populations.

## Limitation

This study has several limitations that warrant consideration. First, its cross-sectional design limits the ability to establish causal inferences regarding the observed associations between sociodemographic determinants and active ageing behaviors. Future longitudinal and cohort studies are needed to clarify temporal relationships and causal pathways. Second, reliance on self-reported questionnaires may have introduced recall and social desirability biases, particularly in relation to sensitive health behaviors such as smoking and alcohol consumption. Third, the study sample was restricted to older adults residing in disaster-prone subdistricts of Bangkok. While this focus enhances contextual relevance, it constrains the generalizability of findings to rural or less hazard-prone populations with differing sociocultural and environmental contexts. Fourth, the exclusion of exercise and oral care from the validated model, due to low factor loadings, indicates potential measurement limitations. Refinement of instruments or development of context-specific indicators may be necessary to more accurately capture these domains in urban ageing populations. Finally, although the GSEM framework enabled the integration of sociodemographic predictors, unmeasured contextual variables such as access to healthcare infrastructure, household income, and social capital were not accounted for and may further influence active ageing trajectories. In addition, one standardized factor loading slightly exceeded 1.0. Although this phenomenon may occur in CFA models using highly reliable composite indicators and robust estimation procedures, the estimate should be interpreted cautiously and may reflect scaling characteristics of the composite measures rather than substantive over-identification of the latent construct. Addressing these limitations will be critical for advancing the robustness and external validity of future research. Although preliminary diagnostics supported the suitability of the data for CFA and GSEM, more extensive assessments of multivariate normality could be explored in future studies. Therefore, the findings should be interpreted as supportive rather than conclusive evidence of the proposed factor structure, and interpretations related to environmental and infrastructural influences should be regarded as contextual hypotheses rather than empirically tested mechanisms. In particular, interpretations related to environmental vulnerability, infrastructural barriers, and service disruptions should be considered theoretically informed contextualizations rather than empirically verified mechanisms within the present dataset. Additionally, although one standardized factor loading slightly exceeded unity, this may occur in CFA models employing highly reliable composite indicators and robust estimation procedures. Future studies may consider item-level modeling or alternative estimation approaches to further examine the stability and generalizability of this parameter. Furthermore, the CFA was conducted using domain-level composite scores rather than item-level indicators. Although this strategy improved model stability and reduced parameter complexity, it constrained the ability to evaluate individual item-factor relationships and to formally test measurement invariance across demographic subgroups. Future research employing item-level modeling may provide a more granular psychometric assessment of the active ageing construct.

## Conclusion

This study provides empirical support for a four-domain representation of active ageing behaviors among older adults in disaster-prone urban communities, based on confirmatory factor analysis and structural modeling. The CFA results confirmed excellent model fit, identifying substance avoidance and stress management as the most salient behavioral domains, with self-care and dietary practices contributing additional structural validity. The exclusion of exercise and oral care due to weak factor loadings underscores the need for contextual refinement of measurement tools to ensure applicability across diverse urban environments.

The GSEM analysis extended these findings by demonstrating that active ageing was significantly associated with both individual health behaviors and sociodemographic determinants, including sex, education, chronic disease, and living arrangements. These results affirm that active ageing appears to reflect both a behavioral construct and a socially embedded process, shaped by individual practices as well as structural conditions. Furthermore, the study highlights the contextual constraints associated with environmental vulnerabilities, such as recurrent flooding, air pollution, and heat stress, on the sustainability of health behaviors in urban settings.

Taken together, these findings underscore the necessity of embedding behavioral resilience into active ageing frameworks and of aligning behavioral promotion with structural and environmental interventions. By addressing disparities arising from social and environmental determinants, policymakers and practitioners can design more inclusive, context-specific, and sustainable strategies to promote healthy ageing. However, these conclusions should be interpreted in light of the study’s cross-sectional design, urban hazard-specific sample, and the use of ordinal self-reported measures, which may limit broader generalization beyond similar contexts. The evidence generated by this study contributes directly to strengthening the knowledge base for health promotion and disaster preparedness policies tailored to vulnerable older populations in rapidly urbanizing, hazard-prone contexts.

## Supplementary Information

Supplementary Information.

## Data Availability

The dataset used in this study is not publicly available due to institutional restrictions but may be obtained from the corresponding author upon reasonable request and with permission from the Navamindradhiraj University Research Ethics Review Committee. In addition, summary statistics and correlation matrices necessary for replication of the analytical approach are fully reported within the manuscript.

## Abbreviations

CFA: Confirmatory factor analysis
CFI: Comparative fit index
GSEM: Generalized structural equation modeling
RMSEA: Root mean square error of approximation
SEM: Structural equation modeling
SRMR: Standardized root mean square residual
TLI: Tucker-Lewis index
WHO: World Health Organization
