# Supplementary material for: Active ageing behaviors among urban older adults in disaster-prone communities using confirmatory factor analysis of health behavior constructs
Source: Sci Rep. 2026 Apr 6;16:16373. doi: 10.1038/s41598-026-46240-3 (PMC13212957; doi:10.1038/s41598-026-46240-3)
Supplement: Supplementary file 1 — Supplementary Information. [file 41598_2026_46240_MOESM1_ESM.docx]

**Questionnaire for the Development and Promotion of Active Ageing Among Older Adults: Self-Worth, Health Literacy, and Quality of Life During Disaster Situations**

**This questionnaire comprises two sections, with the current excerpt including:**

**Section 1:** Personal Information

**Section 2:** Assessment of Active Ageing in Disaster Contexts

**Pre-Screening Questions**

1. Are you 60 years of age or older?

🞏 Yes → Proceed to the questionnaire

🞏 No → End of questionnaire

1. Which subdistrict do you currently reside in?

🞏 Dusit

🞏 Wachiraphayaban

🞏 Suan Chitlada

🞏 Si Yaek Mahanak

🞏 Nakhon Chai Si Road

🞏 None of the above → End of questionnaire

**Section 1: Personal Information**

Instruction: Please respond by ticking the appropriate boxes or filling in the blanks as applicable.

1. Current age: ______ years
2. Gender:
   🞏 Male

🞏 Female

1. Marital status:
   🞏 Single

🞏 Married/Registered/Co-habiting
🞏 Divorced

🞏 Widowed

🞏 Separated

1. Current family structure:
   🞏 Nuclear family

🞏 Extended family

1. Whom do you currently live with?
   🞏 Alone

🞏 With spouse

🞏 With spouse and children
🞏 With children

🞏 Others (please specify): ___________

1. Highest level of education attained:
   🞏 No formal education

🞏 Primary education
🞏 Lower secondary education

🞏 Upper secondary or equivalent
🞏 Vocational diploma or equivalent

🞏 Bachelor's degree
🞏 Postgraduate degree

🞏 Others (please specify): _________

1. Do you have any chronic illnesses?
   🞏 None

🞏 Yes (please specify): ___________

1. What is your current social role or community status?
   🞏 Village Health Volunteer (VHV)

🞏 Village headman
🞏 Community wisdom leader

🞏 Community member
🞏 Volunteer in community project

🞏 Others (please specify): ___________

1. Are you currently employed?
   🞏 Unemployed

🞏 Employed (please specify): ___________

**Section 2: Assessment of Active Ageing During Disaster Situations**

Instruction: This section aims to assess your behaviors and health-related practices defined here as indicators of "Active Ageing" in disaster contexts. It is divided into two parts: **Part 1**: General health behaviors (17 items), **Part 2**: Preventive behaviors against communicable or epidemic diseases (5 items) Total: 22 items. Please tick the box that best reflects your actual behavior.

**Part 1: General Health Behaviors**

| **Behavioral Indicator** | | **Average frequency per week** | | | | |
| --- | --- | --- | --- | --- | --- | --- |
|  |  | **6-7 days** | **4-5 days** | **3 days** | **1-2 days** | **Never** |
| **Dietary Habits (Food)** | | | | | | |
| 1. | Consumes at least 0.5 kg of unsweetened fresh fruits and vegetables per day |  |  |  |  |  |
| 2. | Frequently consumes repetitive or monotonous meals |  |  |  |  |  |
| 3. | Eats high-fat foods (e.g., fried items, coconut milk curries, fatty meats) |  |  |  |  |  |
| 4. | Eats sweet snacks (e.g., candies, syrup-based desserts, sugary fruits) |  |  |  |  |  |
| 5. | Drinks sweetened beverages (e.g., carbonated drinks, colored syrup-based drinks) |  |  |  |  |  |
| 6. | Consumes salty or fermented foods, or adds extra fish sauce |  |  |  |  |  |

| **Behavioral Indicator** | | **Average frequency per week** | | | | |
| --- | --- | --- | --- | --- | --- | --- |
|  |  | **6-7 days** | **4-5 days** | **3**  **days** | **1-2 days** | **Never** |
| **Physical Activity (Exercise)** | | | | | | |
| 7. | Engages in continuous exercise (~30 minutes) until experiencing sweating or fatigue |  |  |  |  |  |
| 8. | Engages in sustained daily physical activity  (~30 minutes) from routine tasks (e.g., walking, housework) |  |  |  |  |  |
| **Stress Management (No stress)** | | | | | | |
| 9. | Experiences loss of appetite or overeating due to stress |  |  |  |  |  |
| 10. | Has difficulty sleeping, fragmented sleep, or excessive sleep |  |  |  |  |  |
| **Substance Use (No smoking and alcohol)** | | | | | | |
| 11. | Smokes cigarettes or uses tobacco products |  |  |  |  |  |
| 12. | Is exposed to secondhand smoke from individuals nearby |  |  |  |  |  |
| 13. | Consumes alcoholic beverages (e.g., beer, wine, spirits, traditional liquors) |  |  |  |  |  |
| 14. | Consumes traditional herbal liquors (infused spirits) |  |  |  |  |  |
| **Oral Health (Dental care)** | | | | | | |
| 15. | Brushes teeth every morning and before bedtime |  |  |  |  |  |
| 16. | Uses dental floss when food is lodged between teeth |  |  |  |  |  |
| 17. | Bites hard objects (e.g., eyeglass frames, pens, sewing needles, plastic packaging) |  |  |  |  |  |

**Part 2: Preventive Behaviors Against Communicable and Epidemic Diseases (Past 3 Months)**

Instruction: Please indicate how frequently you engaged in the following preventive behaviors over the past 3 months.

| **Frequency** | **Definition** |
| --- | --- |
| Always | You performed the action 100% of the time |
| Often | You performed the action more than 70-99% of the time |
| Sometimes | You performed the action approximately 50-69% of the time |
| Rarely | You performed the action less than 49% of the time |
| Never | You never performed the action |

| **Preventive Behavior** | | **Frequency of Practice** | | | | |
| --- | --- | --- | --- | --- | --- | --- |
|  |  | **Always** | **Often** | **Sometimes** | **Rarely** | **Never** |
| 18. | Share personal items (e.g., spoons, glasses, towels) with others |  |  |  |  |  |
| 19. | Eat meals together with others |  |  |  |  |  |
| 20. | Wash hands with soap and water or use alcohol-based hand sanitizer after touching public surfaces (e.g., stair railings, doorknobs, elevator buttons) |  |  |  |  |  |
| 21 | Wear cloth or surgical face masks |  |  |  |  |  |
| 22. | Maintain physical distance of at least 1 meter (or one arm’s length) from others |  |  |  |  |  |
